# Analysis of C5 palsy in cervical myelopathy with massive anterior compression following laminoplasty

**DOI:** 10.1186/s13018-018-0715-3

**Published:** 2018-02-02

**Authors:** Guangdong Chen, Yifan Wang, Zhidong Wang, Ruofu Zhu, Huilin Yang, Zongping Luo

**Affiliations:** Department of Orthopedics of First Affiliated Hospital and Orthopedic Institute of Soochow University, 188 Shizi St, Suzhou, Jiangsu Province 215006 China

**Keywords:** Myelopathy, Laminoplasty, Result, C5 palsy

## Abstract

**Background:**

Little data is available about comparison of the incidence and clinical characteristics of the C5 palsy between patients of cervical myelopathy with occupying ratio greater than 50% and those with occupying ratio less than 50% following laminoplasty.

**Methods:**

One-hundred eighteen patients with cervical myelopathy who underwent open door laminoplasty were reviewed in this study. The patients were divided into two groups: group A comprising 55 patients with an anterior occupying ratio greater than 50% and group B comprising 63 patients with an anterior occupying less than 50%. Clinical and radiological outcomes were assessed between two groups.

**Results:**

No statistically difference was found in preoperative Japanese Orthopedic Association (JOA) score of both groups (10.7 ± 1.7 in group A vs 10.9 ± 1.1 in group B, *P* > 0.05). Improvements in postoperative JOA score were achieved, and there was a statistical difference (14.0 ± 1.4 in group A vs 14.8 ± 0.9 in group B, *P* < 0.05). Group A had a lower rate of recovery than group B (*P* < 0.05). Totally, 12 of 118 (10.2%) patients developed the C5 palsy postoperatively. C5 palsy occurred in 3 of 63 patients in the group B compared with 9 of 55 in the group A. Statistically significant difference was found in the incidence of C5 palsy between the two groups (*P* < 0.05). Furthermore, patients in group A required significantly longer recovery periods than group B. Both preoperative and postoperative MRI presented more levels of T2 high-signal lesion in group A than group B. The degree of posterior shift of the cord after posterior decompression in group A was less than group B (*P* < 0.05).

**Conclusions:**

Patients with a high degree of anterior compression have higher risk of C5 palsy than those with a relative low degree of anterior compression.

## Background

The number of patients with cervical myelopathy needing surgery has increased proportionately with the increasing age of the population. Laminoplasty is commonly accepted for the management of cervical myelopathy resulting from spondylotic disease and ossification of the posterior longitudinal ligament. Actually, encouraging results are obtained from the procedure in the literature [1, 2]. However, spinal specialists and patients have been plagued by segmental motor paralysis of C5, so-called C5 palsy, as one of the clinically significant complications postoperatively [3, 4].

The C5 palsy is defined as the paralysis of the deltoid and/or biceps brachii muscles after surgery without any deterioration of myelopathy symptoms. C5 palsy may add a significant burden upon patients’ quality of life. The affected patients suffer from debilitating symptoms and are often dissatisfied with their surgery. The incidence of postoperative C5 palsy is 4.5% when open-door laminoplasty is performed in a systematic review by Gu et al. [4]. The exact etiology remains uncertain, but it has been attributed to pathologies of the spinal cord or nerve root. Cervical myelopathy with massive anterior compression may be associated with more risk of spinal cord injury or nerve root injury following laminoplasty. Therefore, the author wonders whether the incidence of C5 palsy would increase in cervical myelopathy with massive anterior compression following laminoplasty. To date, there is a lack of detailed report about C5 palsy in cervical myelopathy with massive anterior compression following laminoplasty.

The purpose of this study is to compare the incidence and clinical characteristics of the C5 palsy between patients of cervical myelopathy with massive anterior compression and those without massive anterior compression following open-door laminoplasty.

## Methods

### Patients

**Fig. 1:**
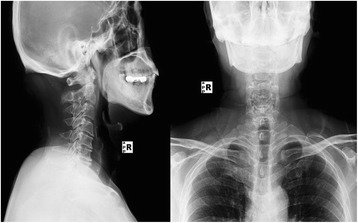
Preoperative x-ray of a 62-year-old female patient with cervical myelopathy

**Fig. 2:**
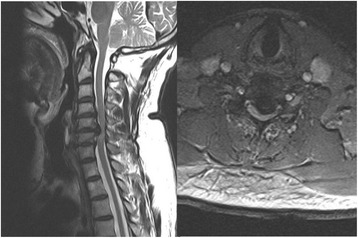
Preoperative magnetic resonance imaging showing at least three level anterior compressions of spinal cord with an occupying ratio more than 50%

A retrospective analysis was performed on 118 patients with cervical myelopathy who underwent open door laminoplasty between January 2005 and December 2011. These patients had undergone no previous cervical surgery. All patients maintained cervical lordosis. Patients with cervical myelopathy due to spinal cord tumor were also excluded. Clinical diagnosis was made by physical examination, plain radiography, computed tomography, and magnetic resonance imaging. All patients presented with cervical myelopathy characterized by exaggerated tendon jerk in their extremities and spasticity due to multilevel spinal stenosis without radiculopathy. The study was carried out with the approval of the ethics committee of our institution (Figs. 1 and 2).

### Surgical procedures

**Fig. 3:**
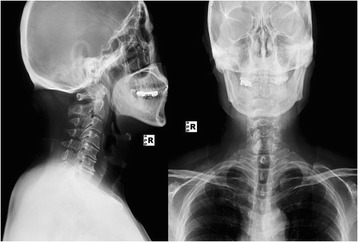
Postoperative x-ray showing laminoplasty with suture fixation keeping the lifted lamina open

**Fig. 4:**
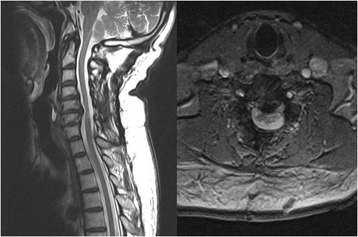
Postoperative magnetic resonance imaging demonstrating extensive decompression

Expansive open door laminoplasty, as described by Hirabayashi et al. [5], was performed, and decompression was extended from C3 to C7 in all patients. Spinous processes and supraspinous ligaments were retained. The drilled gutter on the lateral margin of the lamina was first completed on the open side. Drilling of the bony gutter on the hinge side was slightly lateral compared with that on the open side to prevent the lamina from becoming unstable or dislodging. The elevated laminae were secured with sutures or plate. Patients were instructed to wear a neck collar for 3 to 4 weeks postoperatively (Figs. 3 and 4).

### Clinical evaluation

The neurologic evaluation was graded using the scale devised by the Japanese Orthopedic Association (JOA). The rate of recovery, which indicated the degree of normalization after surgery, was calculated using Hirabayashi’s formula: (postoperative score − preoperative score) × 100 / (17 [full score] – preoperative score). The incidence of C5 palsy was examined. C5 palsy was defined as weakness of grade 4 or less of the key muscles in the upper extremity by manual muscle test (MMT) without any deterioration of myelopathic symptoms after surgery.

### Radiological evaluation

Cervical sagittal alignment was determined by the Cobb angle between C2 and C7 in lateral cervical spine x-rays. The presence of preoperative signal intensity change in the cord was evaluated on sagittal view T2-weighted MRI. The level with massive anterior compression and posterior shift of spinal cord was measured from MRI. The distance between the posterior borderline of vertebral body to the anterior edge of the spinal cord was measured at each laminoplasty level in midsagittal images of T2-weighted MRI. The cord shift distance was defined as the difference between preoperative and 1 year postoperative measured values. The average value of cord shift distance from C3 to C7 was obtained for a given patient. The occupancy ratio of compressive mass is defined as the maximal thickness of the compressive mass divided by the anteroposterior diameter of the bony spinal canal in the same segment.

**Fig. 5:**
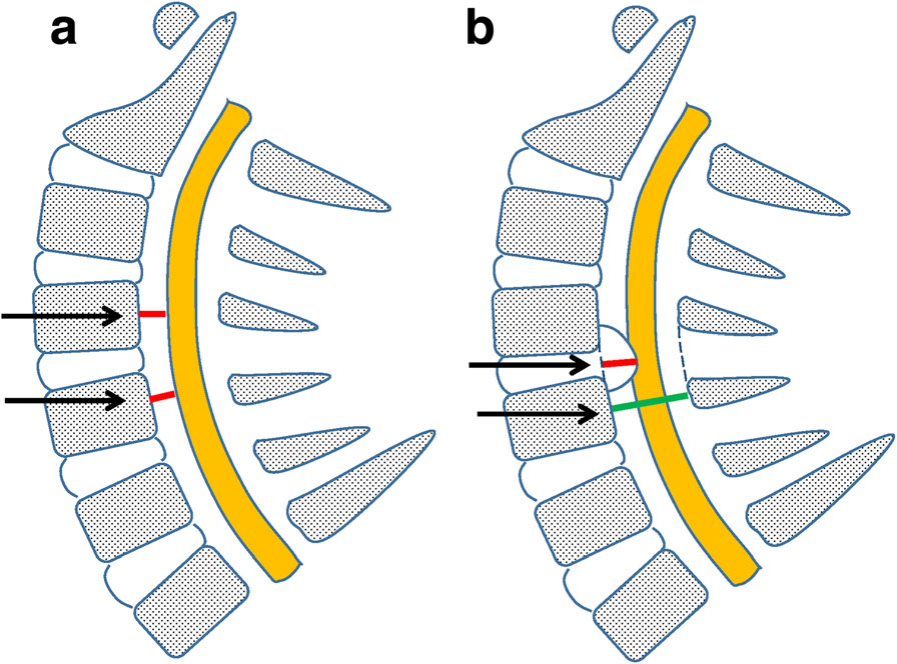
Schematic diagram of measurement. **a** The red line showing the distance between the posterior borderline of vertebral body to the anterior edge of the spinal cord. Shift distance = postoperative value − preoperative value. **b** The red line showing the maximal thickness of the compressive mass, while the green line showing the anteroposterior diameter of the bony spinal canal. The occupancy ratio of compression mass = length of red line/length of green line

An anterior occupying ratio greater than 50% is defined as massive anterior compression. The 118 patients were divided into two groups: group A comprising 55 patients with an anterior occupying ratio greater than 50% and group B comprising 63 patients with an anterior occupying less than 50% (Fig. 5).

### Statistical analysis

Data were presented as means ± standard deviation. A *t* test with Welch correction was used for statistical analysis of the difference in the mean values between the two groups. The *χ*^2^ test for independence was used to compare the incidence of C5 palsy between two groups. A *P* value < 0.05 was considered statistically significant.

## Results

### Clinical results

Group A consisted of 45 males and 10 females with mean age of 59 years (range from 40 to 84). Group B included 49 males and 14 females with mean age of 57 years (range from 42 to 74). Mean duration of symptoms before surgery was 18.5 ± 11.9 months in group A, which was more than 14.7 ± 7.6 months in group B (*P* < 0.05). Average follow-up time for group A was 37.1 ± 15.7 months (range from 18 to 84), while for group B was 36.6 ± 15.1 months (range from 18 to 82). No significant difference was found in the other demographic data between the two groups (Table 1).

**Table 1:** Patient general demographic data

	Group A	Group B	*P*
Age (years)	59.4 ± 10.5	57.7 ± 8.2	0.351
Mean duration of symptoms (months)	18.5 ± 11.9	14.7 ± 7.6	0.043*
Operative time (min)	156.9 ± 40.7	149.1 ± 30.9	0.241
Time of follow-up (months)	37.1 ± 15.7	36.6 ± 15.1	0.858
Blood loss (ml)	327.8 ± 132.0	308.4 ± 108.8	

Values are means ± SD

*Statistically significant, *P* < 0.05

Improvements in mean JOA score were achieved in both groups (mean JOA score improved from 10.7 ± 1.7 to 14.0 ± 1.4 in group A and from 10.9 ± 1.1 to 14.8 ± 0.9 in group B). The rate of recovery was 51.4 ± 25.7 in group A and 64.8 ± 11.9 in group B. Group A had a lower rate of recovery than group B (*P* < 0.05) (Table 2).

**Table 2:** JOA score and C5 palsy at the final follow-up

	Group A	Group B	*P*
Pre JOA score	10.7 ± 1.7	10.9 ± 1.1	0.388
Post JOA score	14.0 ± 1.4	14.8 ± 0.9	0.000*
JOA recovery rate (%)	51.4 ± 25.7	64.8 ± 11.9	0.001*
C5 palsy on open side	8/55	3/63	0.069
C5 palsy on hinge side	6/55	1/63	0.033*
Total C5 palsy	9/55	3/63	0.038*

Values are means ± SD

*Statistically significant, *P* < 0.05

JOA indicates Japan Orthopedic Association

Totally, 12 of 118 (10.2%) patients developed the C5 palsy postoperatively. Of the 12 patients, 5 had ossification of the posterior longitudinal ligament (OPLL) and 7 had CSM. The C5 palsy developed between 2 days and 6 weeks postoperatively in both groups. Seven cases occurred unilaterally and five cases presented bilaterally. One case experienced paralysis on the hinge side and six cases was on the open side. C5 palsy occurred in 3 of 63 patients in group B compared with 9 of 55 in group A. Statistically significant difference was found in the incidence of C5 palsy between the two groups (*P* < 0.05). Group A had higher risk of C5 palsy than group B. Furthermore, nine cases of MMT ≤ 2 in group A experienced more severe paralysis than 3 cases of MMT ≥ 3 in group B. No patient with C5 palsy needed further operation. All patients were treated conservatively with rest, intravenous corticosteroids for 2 or 3 days, rehabilitation of muscle strength, and physiotherapy. Spontaneous recovery was observed in three cases of group B at 5, 7, and 10 months postoperatively, respectively. However, patients in group A required significantly longer recovery periods than those in group B. Five of nine patients took more than 12 months to recover. Four of nine patients have not fully recovered at the latest follow-up.

### Radiographic results

No significant differences of cervical alignment were found between two groups preoperatively. Fourteen of 55 patients in group A and 12 of 63 patients in group B showed OPLL signs. Mean C2–C7 lordotic angle decreased from 15.7 ± 9.8° to 14.0 ± 10.1° in the group A and from 17.8 ± 8.3° to 15.0 ± 8.1° in the group B, indicating no significant difference between the groups in postoperative kyphotic change. However, both preoperative and postoperative MRI presented more levels of T2 high-signal lesion in group A than group B. The preoperative MRI showed the vertebral level of maximal occupancy ratio of compression in patients with C5 palsy: C3–C4 in three patients, C4–C5 in six patients, and C5–C6 in three patients. The degree of posterior shift of the cord after posterior decompression in group A with a high degree of anterior compression was less than group B with a relative low degree of anterior compression (*P* < 0.05) (Table 3).

**Table 3:** Radiological data between two groups at the final follow-up

	Group A	Group B	*P*
OPLL	14/55	12/63	0.407
Preoperative cervical lordosis (°)	15.7 ± 9.8	17.8 ± 8.3	0.217
Postoperative cervical lordosis (°)	14.0 ± 10.1	15.0 ± 8.1	0.538
Preoperative T2 high-signal intensity	24/55	12/63	0.004*
Postoperative T2 high-signal intensity	39/55	14/63	0.000*
Mean occupying rate%	62.3 ± 9.5	44.5 ± 3.1	0.000*
Mean spinal cord posterior shift (mm)	2.08 ± 0.77	2.81 ± 0.66	0.000*

Values are means ± SD

*Statistically significant, *P* < 0.05

OPLL indicates ossification of the posterior longitudinal ligament

### Univariate and multivariate logistic regression analysis for risk factors of C5 palsy

A logistic regression was performed to identify the relevant risk factors of C5 palsy following laminoplasty. Univariate and multivariate analyses based on gender, age (classified by generation), group, OPLL, and preoperative T2 high-signal intensity (C4/5) were carried out, which demonstrated group to be significant risk factor for developing C5 palsy (OR 0.181 [0.038–0.867], *P* < 0.05). Factors including gender, age, OPLL, and preoperative T2 high-signal intensity (C4/5) did not significantly affect the incidence of C5 palsy following laminoplasty (Table 4).

**Table 4:** Binary logistic regression analysis for risk factors of C5 palsy

	Univariate		Multivariate	
	OR (95%CI)	*P*	OR (95%CI)	*P*
Gender	0.741 (0184–2.979)	0.673	0.523 (0.116–2.362)	0.399
Age				
< 50	Reference			
51–59	2.073 (0.225–19.093)	0.520	2.018 (0.201–20.228)	0.551
60–69	2.500 (0.270–23.124)	0.419	3.458 (0.326–36.644)	0.303
> 69	1.214 (0.069–21.217)	0.894	0.745 (0.039–14.152)	0.845
Group	0.256 (0.065–0.998)	0.050	0.181 (0.038–0.867)	0.032*
OPLL	1.203 (0.301–4.810)	0.794	1.167 (0.278–4.903)	0.833
Preoperative T2 high-signal intensity (C4/5)	1.728 (0.509–5.863)	0.380	0.928 (0.237–3.631)	0.914

*Statistically significant, *P* < 0.05

OR indicates odds ratio, CI indicates confidence interval, OPLL indicates ossification of the posterior longitudinal ligament

## Discussion

Postoperative segmental motor paralysis of C5, so-called C5 palsy, has reported as one of the relatively uncommon but serious clinical complications after decompression surgery for cervical myelopathy [3, 4, 6]. The incidence of C5 palsy varies from 0 to 30.0% in different studies [7]. In a multicenter study, Imagama et al. reviewed 1858 patients following laminoplasty and identified a relatively low incidence (2.3%) due to rigid definition of C5 palsy with an MMT score of 2 or less [8]. A recent systematic review reported that incidence of postoperative C5 palsy is 4.5% after laminoplasty [4]. In this study, the overall rate of C5 palsy was 4.8% in group B, which is comparable with the previously reported values [3, 4].

The precise etiology of C5 palsy remains obscure in the literature, including iatrogenic, reperfusion injury, and C5 tethering after posterior decompression [8–13]. These proposed etiologies are mainly associated with pathologies of the spinal cord or nerve root.

Originally, intraoperative iatrogenic injury to the C5 nerve root has been proposed as the etiology of C5 palsy. The C5 palsy was attributed to the heat injury of nerve root generated by a high-speed diamond burr during surgery [8]. A following study validated this finding in an experimental porcine model [14]. Nevertheless, Nakamae et al. did not detect abnormal changes on transcranial electric motor-evoked potential monitoring even in those patients who developed postoperative C5 palsy during cervical laminoplasty [15]. As a result, the etiology of intraoperative iatrogenic injury could not explain various cases with C5 palsy, in which the C5 palsy typically appeared for several days after surgery. Sasai et al. argued that high-speed drills must come much closer to nerve root than any other procedure during foraminotomy, but the procedure did not increase the incidence of postoperative C5 palsy [16]. Therefore, the thermal damage to nerve roots during creation of bony gutter is assumed not to induce C5 palsy.

In this study, more levels of T2 high-signal lesion presented in group A than group B postoperatively. This finding can be explained by local reperfusion after decompression of a chronic compressive lesion of the cervical cord. In addition, the patients of group A with high occupying ratio had more risk of experiencing serious reperfusion following decompression procedure. Reperfusion injury after decompression procedure was proposed as an etiology of the paralysis [17, 18]. Local reperfusion of the spinal cord may cause cellular damage. Chiba et al. suggested the cause of C5 palsy to be reperfusion injury of the spinal cord after surgery [17]. Previous reports have also suggested that a high-signal intensity zone is seen more often on MRI at the paralyzed level [15, 19, 20]. The similar phenomenon was presented in our study, which could partially explain the higher incidence of C5 palsy in group A compared with group B. However, there is still half of the patients exhibiting unilateral paralysis, which could not be fully explained by only spinal cord injury.

The tethering effect on the nerve root induced by excessive posterior shift of the spinal cord after laminoplasty is another hypothesis put forward by a number of authors [9, 10, 13]. However, the degree of posterior shift of the cord after posterior decompression in group A with a high degree of anterior compression was less than group B with a relative low degree of anterior compression in our study, but incidence of C5 palsy is higher in group A than in group B. The paradox could be explained as follows. The cord shift distance was measured between the posterior borderline of vertebral body to the anterior edge of the spinal cord. When the anterior compressive mass is very large, and the spinal cord is compressed seriously before surgery, the anteroposterior diameter of spinal cord might change greatly after surgery, but the anterior edge of spinal cord may still tightly contact with the compressive factor resulting in less distance of posterior shift. Nevertheless, the nerve root became more tightened due to expansion of compressive spinal cord after surgery, which might contribute to the impairment of nerve root [21, 22]. If we evaluate the mid-point of the spinal cord, the result would be changed. However, the changed result would bring another paradox (patients with more posterior shift showed worse neurological recovery).

Spontaneous recovery of C5 palsy has been reported in various literatures with conservative treatment [6, 8, 13, 23]. In our study, patients with higher occupying rate experienced more severe paralysis. The severely paralyzed cases of group A required significantly longer recovery periods than the mild cases of group B. Additionally, four of nine patients in group A have not fully recovered at the latest follow-up. The results indicated that the good prognosis did not occur in all patients with C5 palsy.

Previous investigators have attempted to either identify or prevent the occurrence of C5 palsy intraoperatively [24, 25]. Nakamae et al. used intraoperative spinal cord monitoring with transcranial electric motor-evoked potentials to investigate segmental motor paralysis [15]. However, no abnormal finding was found in those patients who developed postoperative C5 palsy. The incidence of the C5 palsy in group A was 16.4%, which is higher than the rate of 4.5% reported in the literature [4]. By comparisons of clinical parameters, we find patients with a high anterior occupying ratio, which is a risk factor of upper extremity palsy following laminoplasty. We should keep in mind of postoperative segmental motor paralysis when patients with high occupation are treated by laminoplasty. A recommended prophylactic C4/C5 foraminal decompression and intravenous corticosteroids could be considered in those with an anterior occupying ratio greater than 50% during the procedure.

## Conclusion

Patients with a high degree of anterior compression have higher risk of C5 palsy than those with a relative low degree of anterior compression. Patients with high occupation experience more severe C5 palsy and longer recovery times from palsy.

## Abbreviations

CSM: Cervical spondylotic myelopathy
JOA: Japanese Orthopedic Association
MMT: Manual muscle test
OPLL: Ossification of the posterior longitudinal ligament
